# Preventive Action of Beta-Carotene against the Indoxyl Sulfate-Induced Renal Dysfunction in Male Adult Zebrafish via Regulations of Mitochondrial Inflammatory and β-Carotene Oxygenase-2 Actions

**DOI:** 10.3390/biomedicines11102654

**Published:** 2023-09-27

**Authors:** Arunachalam Muthuraman, Abu Sadat Md. Sayem, Sakthiganapathi Meenakshisundaram, Nemat Ali, Sheikh F. Ahmad, Abdullah F. AlAsmari, Shamama Nishat, Khian Giap Lim, Yamunna Paramaswaran

**Affiliations:** 1 Pharmacology Unit, Faculty of Pharmacy, AIMST University, Semeling, Bedong 08100, Kedah, Malaysia; 2 School of Pharmacy, Sri Balaji Vidyapeeth, Pillaiyarkuppam, Pondicherry 607402, India; 3 Department of Pharmacology and Toxicology, College of Pharmacy, King Saud University, P.O. Box 2457, Riyadh 11451, Saudi Arabia; 4 Comprehensive Cancer Center, Wexner Medical Centre, Ohio State University, Columbus, OH 43210, USA

**Keywords:** adenosine triphosphate, carotenoids, glutathione peroxidase-1, hematoxylin staining, intestinal flora, reduced glutathione, tryptophan

## Abstract

Indoxyl sulfate (IS) is a metabolic byproduct of indole metabolism. IS readily interacts with the mitochondrial redox metabolism, leading to altered renal function. The β-carotene oxygenase-2 (BCO2) enzyme converts carotenoids to intermediate products. However, the role of β-carotene (BC) in IS-induced renal dysfunction in zebrafish and their modulatory action on BCO2 and mitochondrial inflammations have not been explored yet. Hence, the present study is designed to investigate the role of BC in the attenuation of IS-induced renal dysfunction via regulations of mitochondrial redox balance by BCO2 actions. Renal dysfunction was induced by exposure to IS (10 mg/L/hour/day) for 4 weeks. BC (50 and 100 mg/L/hour/day) and coenzyme Q10 (CoQ10; 20 mg/L/hour/day) were added before IS exposure. BC attenuated the IS-induced increase in blood urea nitrogen (BUN) and creatinine concentrations, adenosine triphosphate (ATP), and complex I activity levels, and the reduction of renal mitochondrial biomarkers, i.e., BCO2, superoxide dismutase-2 (SOD2), glutathione peroxidase-1 (GPX1), reduced and oxidized glutathione (GSH/GSSG) ratio, and carbonylated proteins. Moreover, renal histopathological changes were analyzed by the eosin and hematoxylin staining method. As a result, the administration of BC attenuated the IS-induced renal damage via the regulation of mitochondrial function.

## 1. Introduction

Indoxyl sulfate (IS, 3-hydroxy indole) is a major metabolic product of indole metabolism [1]. Indole is present in various “cruciferous” vegetables like Brussels sprouts, bok choy, turnips, broccoli, cabbage, and kale [2]. The gut microbiome plays a major role in indole metabolism to control various disease manifestations [3]. Chemically, IS is called 3-indoxyl sulfuric acid. IS is a metabolic product of dietary L-tryptophan and interacts with various cellular events via the activation of a sulfur moiety of cellular proteins [4]. However, the metabolic process of indole in intestinal microflora readily converts the indole and indole-containing amino acid, i.e., tryptophan, to IS (uremic toxic molecule), which is normally excreted through urine [5,6]. However, in chronic kidney disease (CKD) conditions, a decrease in the renal clearance of IS via renal filtration leads to the accumulation of IS in the blood circulation in CKD patients [7,8]. Furthermore, IS interacts with mitochondrial redox principles and causes renal dysfunction [9]. In addition, the inner membrane of mitochondria has a BCO2 enzyme (EC 1.14.99), and it helps in catalytic conversion of carotenoids [10]. Experimental evidence revealed that the deficiency of BCO2 contributes to the progression of renal dysfunction via the alteration of mitochondrial membrane potential [11]. Moreover, the toxic mechanism of IS in the progression of CKD also accelerates the production of oxidative stress due to the deficiency of free radical defense enzymes like SOD2, and GPX1; and a decrease in the GSH levels leads to reduction in the ATP contents [12,13]. Extreme levels of IS cause endothelial dysfunction and renal injury [14,15]. The metabolomics analysis of the IS effect revealed that it causes CKD in zebrafish via elevation of IS in plasma [16] and in mice [17]. The natural accumulation of IS is known as an indicator of the progression of CKD [8]. In CKD patients, it indicates that the metabolic accumulation of IS worsens the CKD condition [18]. Moreover, it is also a chance to produce cardiovascular dysfunction and death [19]. The renal dialysis process has also failed to manage the IS-associated CKD improvement due to the binding nature of IS to albumin [20]. Furthermore, numerous studies on IS-associated CKD were investigated with an evaluation of metabolomics and genomic analysis [16,21]. Furthermore, multiple newer molecular pathways and pathological concepts remain to be explored. Hence, it is essential for the evaluation of uremic toxin pathway regulation for the management of renal disorders.

Clinically available medicines, i.e., antibiotics, pain killers, antidiabetics, proton pump inhibitors, and antihyperlipidemic agents, are known to cause renal injury and endothelial dysfunction [22]. Hence, these agents need to be avoided, and dose adjustments are essential for CKD patients [23,24]. IS is one of the novel metabolic uremic toxins. Some of the nephroprotective agents are still used with special caution; such agents are co-trimoxazole, isoniazid, azathioprine, prednisolone, cyclophosphamide, and erythropoietin. They are widely used for renal transplantation and other renal disorders [25,26]. However, the chronic usage of these agents for renal disorders remains challenging due to low therapeutic outcomes and other complications [27]. Herbal medicines are a greater promising approach for the management of renal disorders. Some of the herbals are used for renal disorders, like *Boerhavia diffusa* [28], *Anthocleista vogelii*, *Alstonia boonei* [29], Astragalus, Ligusticum, Triptolide, and Rhubarb [30]. Some herbal constituents honokiol [31], isoliquiritigenin, β-sitosterol, apigenin, betulinic acid quercetin, rutin, gallic acid, and catechin [32], are known to produce nephroprotective action via the regulation of oxidative stress mechanism and removal of uremic toxins [33]. Even α-tocopherol, ubiquinol, bilirubin, glutathione, and ascorbic acid including BC are known to provide nephroprotective action via the removal of uremic toxins in CKD patients [34].

BC is one of the natural antioxidants, and it is a vitamin A precursor. It possesses various tissue-protective actions, including renal tissue due to multi-targeted actions [35]. BCO2 is a metabolic key enzyme for various carotenoid metabolisms like lycopene, lutein, and zeaxanthin, including BC in animals [36,37]. Furthermore, a deficiency of BCO2 is known to cause mitochondrial oxidative stress, and mitochondrial fragmentation leads to metabolic dysfunction of BC and causes tissue inflammation [38]. Furthermore, experimentally, BC protects ischemia/reperfusion injury-induced renal injury in rats [39]. Clinical reports have also revealed that a low intake of β carotene worsens the condition of renal tissue functions in CKD patients [40]. Mitochondrial inflammation is a major hallmark of the progression of CKD [41]. However, the role of BC in IS-induced renal dysfunction in zebrafish and their modulatory action on BCO2 and mitochondrial inflammation have not been studied yet. Hence, the present study was designed to investigate the role of BC in the regulation of IS-induced renal dysfunction and mitochondrial inflammation in adult male zebrafish models via BCO2 action.

## 2. Materials and Methods

### 2.1. Animals

The disease-free adult male zebrafish (eight months old) were used in this study. All the zebrafish were maintained in the housing tank (10 L) with potable drinking water. The water was conditioned with an aerator (to supply the dissolved oxygen), thermostatic device (to maintain the water temperature, i.e., 25 ± 0.5 °C), and, i.e., 14:10 h light and dark cycles with suitable automatic timer controller and light system. The 2 weeks acclimatization period was provided to adapt the zebrafish to a newer environmental setup. All the animals were supplied the standard floating food throughout the experimental protocol. The experimental protocol approval number of the zebrafish trial protocol was IAEC/02/04/023.

### 2.2. Drugs and Chemicals

The test compound, i.e., BC, was obtained from palm oil mill effluent (supplied by Palm Oil Mill Sdn. Bhd., Penang, Malaysia). The reference control drug, i.e., CoQ10, was procured from Pharma Nord Sdn Bhd, Kuala Lumpur, Malaysia. 5,5-dithibis(2-nitrobenzoic acid) (DTNB), ethylene glycol-bis(β-aminoethyl ether)-N,N,N′,N′-tetraacetic acid (EGTA), 10 mM of 4-(2-hydroxyethyl)-1-piperazineethanesulfonic acid (HEPES), β-cryptoxanthin, nicotinamide adenine dinucleotide phosphate (NADPH), nicotinamide adenine dinucleotide hydrogen (NADH), protein carbonyl content colorimetric assay kit, and ATP were purchased from Merck Sdn Bhd, Selangor, Malaysia. The SOD2 enzyme-linked immunosorbent assay (ELISA) kit was procured from LSBio, Selangor, Malaysia. Blood urea nitrogen (BUN) and creatinine kits were procured from Biogenix Inc. Pvt. Ltd., Lucknow, India. Protein carbonyl content colorimetric assay kit was procured from Sigma-Aldrich Sdn. Bhd., Petaling Jaya, Malaysia. The mitochondrial complex I activity colorimetric assay kit was procured from Abcam Sdn. Bhd., Kuala Lumpur, Malaysia. All the chemical reagents were used as an analytical grade.

### 2.3. Induction of Renal Dysfunction by IS Exposure

The IS-induced renal dysfunction in zebrafish via elevated oxidative stress was carried out as described by Tang et al. [16]. Further, IS also causes mitochondrial inflammation [42] and uremic endothelial-toxins [14]. Briefly, the renal dysfunction was induced by exposure to IS at the dose of 10 mg/L. The IS solution was freshly prepared daily for induction of renal dysfunction in zebrafish by dissolving it in distilled water. Then fish were placed in the freshly prepared IS solution for 1 h. After 1 h of exposure, animals were returned to their home cage tank. These patterns were repeated for 4 weeks of the study period. At the end of the 4th week, animals were sacrificed for further biomarker and histopathological observations.

### 2.4. Experimental Protocol

Five groups were used in this research work, and each group comprised 22 male adult zebrafish animals (*n* = 22).

⮚ Group 1: This group was employed as a normal control group without exposure to any IS, BC, and CoQ10 agents.
⮚ Group 2: This group was employed as an IS-exposed group (as a negative control group). The induction of renal dysfunction was made as described in the previous sections.
⮚ Groups 3 and 4: This group was employed to exposure to BC doses, i.e., 50 and 100 mg/L/hour/day for 4 weeks in IS-exposed animals, respectively.
⮚ Group 5: This group was employed to expose CoQ10 (20 mg/L/hour/day) for 4 weeks in IS-exposed animals. CoQ10 is a key component of the mitochondrial respiratory chain, and it acts as an endogenous antioxidant [43]. Experimentally, it prevents renal mitochondrial dysfunction and renoprotective actions [44]. Clinically, CoQ10 improves mitochondrial function and attenuates end-stage renal disease [45]. Hence, it is used as a reference drug in this study.

The test compounds, i.e., BC and CoQ10, were exposed 30 min before IS exposure. At the end of the 4 weeks of exposure to IS and BC, all the animals were sacrificed, and renal tissue was isolated and used for the estimation of BCO2, SOD2, GPX1, GSH, carbonylated protein, and ATP concentrations. Furthermore, the renal histopathological changes were analyzed by the eosin and hematoxylin staining method.

### 2.5. Estimation of BUN and Creatinine Concentration

At the end of the study protocol (last days of 4th week), animals were employed for the collection of blood samples by caudal fin junction amputation method as described by Babaei et al. [46]. Briefly, zebrafish were anesthetized with 0.04 mg/mL of MS-222 (ethyl-3-aminobenzoate methanesulfonate) for 3 to 5 min. The amputation of the caudal fin junction was made with fine scissors. Blood was drained out immediately in a micro-centrifuge (0.5 mL capacity) tube and centrifuged at 40× *g* force for 5 min at 11 °C. If the blood sample was too small, then another 1 mm tissue slice was made near the previous amputation and repeated the centrifugation process as above. Then, the fish was taken out and the collected blood sample was centrifuged at 13,700× *g* force for 15 min at 4 °C. The serum samples were used for the estimation of BUN and creatinine concentration by using commercial BUN and creatinine kits (Biogenix Inc., Pvt., Ltd., Lucknow, India).

### 2.6. Estimation of Renal Mitochondrial Biomarkers

Zebrafish renal tissue was collected as described by Gerlach et al. [47]. The tissues were weighed and minced into small pieces. Then, the tissue was homogenized (10% *w*/*v*) with phosphate-buffered saline (PBS) solution (pH~7.4). The renal tissue mitochondria were isolated as a described method by Gross et al. [48]. Briefly, the renal tissue mitochondria were isolated for BCO2 assessment using a semi-frozen mitochondria isolation buffer. It consists of 250 mM of sucrose, 1 mM of EGTA, 10 mM of HEPES, and 0.5% *w*/*v* of bovine serum albumin; the pH was adjusted to 7.4 with diluted potassium hydroxide. The tissue homogenates were centrifuged at 1000× *g* force for 20 min. The homogenate was made with PBS and the pellet was collected and suspended to 5% dextran. The solution was again centrifuged thereafter the mitochondria were resuspended and the pressure cycling step was five times with 10,000 pounds per square inch (psi) for 20 s and at 14.7 psi atmospheric pressure for 5 s with 5% dextran solution to obtain the quality of mitochondria. Pressure cycling steps were performed at 4 °C using the Barocycler 2320EXT device (Pressure BioSciences, Inc., Boston, MA, USA). The clear supernatants and mitochondrial solution were stored at −20 °C until further use for tissue biomarker estimations. Here, sample care was taken to avoid repeated freeze–thaw cycles during the storage and estimation period. The changes in the IS-induced changes of BCO2, SOD2, GPX1, GSH, carbonylated protein, and ATP concentrations were estimated as described in the following sections.

#### 2.6.1. Estimation of Renal Mitochondrial BCO2 Activity

The renal mitochondrial BCO2 activity was estimated as described by Babino et al. [49] with a slight modification of Thomas et al. [50]. Briefly, 100 μL of aliquot was mixed with assay buffer (it contains 100 μL of 0.2% *v/v* Triton X-100) and 10 μM of BCO2 substrate, i.e., β-cryptoxanthin (dissolved in ethanol). The standard plot was assessed by mixing 50 μg of purified BCO2 protein containing β-cryptoxanthin (metabolic precursor of 3-hydroxy-β-12′-carotenal) in 100 μL enzyme assay buffer and 4 to 16 μM of substrate solution. The test and standard solutions were incubated at room temperature (37 °C) in a constant shaking (600 rpm) thermomixer device (Eppendorf, Hamburg, Germany). After 12 min of incubating the solution, the enzyme reaction was stopped by adding 400 μL of acetone, 400 μL of diethyl ether, 100 μL of petroleum ether, and lipophilic compounds. The changes in absorbance were assessed at 420 nm by using a UV-1800 Shimadzu Spectrophotometer (Shimadzu Corporation, Kyoto, Japan). The renal mitochondrial BCO2 activity levels were expressed as nM of BCO2 per mg of protein.

#### 2.6.2. Estimation of Renal Mitochondrial SOD2 Activity

The renal mitochondrial SOD2 activity was assessed by using the ELISA kit method. Briefly, 100 µL of samples were placed in precoated antibody 96 well plates and incubated for 90 min at room temperature (37 °C). The plate was washed, and 100 μL of biotinylated detection Ab was added to each well and incubated at 37 °C for 60 min. The plate was washed three times with washing buffer. Thereafter, 100 μL of Avidin–Horseradish peroxidase conjugate working solution was added and incubated at 37 °C for 30 min. Then, all the wells were washed 5 times with washing buffer and mixed with 90 μL of chromogenic substrate solution (containing 3,3′,5,5′ tetramethylbenzidine dissolved in ethyl acetate) and incubated at 37 °C for 15 min. The enzyme–substrate reaction was terminated by the addition of 50 μL of stop solution. The yellow-color chromogen absorbance changes were estimated spectrophotometrically at a wavelength of 450 nm. The mitochondrial SOD2 activity was expressed as nanogram per milliliter (ng/mL) using a microplate reader (ClaIR™, Photon Etc., Montreal, QC, Canada). The standard plot of the SOD2 activity was prepared using 0.31 to 20 ng/mL. The results were expressed as ng of the SOD2 activity per milligram of protein.

#### 2.6.3. Estimation of Renal Mitochondrial GPX1 Activity

The renal mitochondrial GPX1 activity was estimated as described by Lawrence and Burk [51]. Briefly, the aliquot was sequentially mixed with 1 U/mL of glutathione reductase, 2 mM of glutathione, 0.2 mM of NADPH, 70 μM of t-butyl hydroperoxide, 0.05 mM of ethylenediaminetetraacetic acid, and 10 mM Tris-hydrochloric acid at room temperature. The solution mixture pH was maintained at 7.5. The changes in NADPH absorbance due to GPx activity were estimated at 340 nm by using a UV-1800 Shimadzu Spectrophotometer (Shimadzu Corporation, Kyoto, Japan). One unit of GPX1 enzyme activity was defined as the amount of enzyme catalyzed to oxidation of 1 mM of NADPH substance per minute. The value of renal mitochondrial GPX1 activity was expressed as unit activity per mg protein (U/mg of protein).

#### 2.6.4. Estimation of Renal Mitochondrial GSSG/GSH Ratio

The renal mitochondrial GSH activity was estimated as described by Ellman [52]. Briefly, the renal aliquot was mixed with a 1:1 ratio of 10% *w*/*v* of tri-chloroacetic acid to make the protein precipitation in the aliquot solution. Then, the solution was centrifuged at 4 °C at 134 g force for 10 min. A 0.5 mL volume of supernatant was mixed with 2 mL of disodium hydrogen phosphate (0.3 M) solution and 0.25 mL of Ellman’s reagent (DTNB) 0.001 M solutions. The DTNB was dissolved in 1% *w*/*v* sodium citrate solution. The developed yellow-colored chromogen absorbance changes were estimated at 412 nm by using a UV-1800 Shimadzu Spectrophotometer (Shimadzu Corporation, Kyoto, Japan). The standard plot for GSH was prepared with 10 to 100 micromoles of GSH per mL. The value of renal mitochondrial GSH was expressed as µM/mg of protein. The supernatant was used for quantification of oxidized glutathione disulfide (GSSG), as described previously [53]. Briefly, 1 mL of renal aliquot was mixed with 10 mL of 2-vinyl pyridine solutions. Then, the test tubes were vortexed and incubated at room temperature for 1 h. Color intensity changes were estimated at 412 nm by using a UV-1800 Shimadzu Spectrophotometer (Shimadzu Corporation, Kyoto, Japan). The standard plot for GSSH was prepared with 0 to 50 micromoles of GSSH per mL. The GSSG content was calculated by using the following formula:

$$GSSG=\frac{\left(Total\;Glutathione-GSH\right)}{2}$$

The GSSG/GSH ratio was calculated by using the following formula:

$$GSSG/GSH\;ratio=\frac{\left[GSH\right]}{\left[GSSH\right]}$$

#### 2.6.5. Estimation of Renal Mitochondrial Carbonylated Protein Content

The renal mitochondrial carbonylated protein content was estimated as described in the protein carbonyl content colorimetric assay kit (Sigma-Aldrich (M) Sdn. Bhd., Petaling Jaya, Malaysia). Briefly, 100 μL of renal mitochondrial aliquot was mixed with 100 μL of 2,4-dinitrophenylhydrazine (DNPH) and vortexed followed by incubation at room temperature (37 °C) for 10 min. Then, 30 μL of trichloroacetic acid was mixed in each test tube sample. Crucially, 10 μL of streptozocin and 200 μL of guanidine solution were added and sonicated to resolubilization of proteins. All the samples were subjected to the formation of stable chromogen, i.e., dinitrophenyl (DNP) hydrazine. The absorbance changes of colored chromogen were estimated at 375 nm by using a UV-1800 Shimadzu Spectrophotometer (Shimadzu Corporation, Kyoto, Japan). The standard plot was prepared with 1 to 15 nanomoles of carbonylated protein. The value of renal mitochondrial carbonylated protein content was expressed as nM/mg of protein.

#### 2.6.6. Estimation of Renal Mitochondrial ATP Content

The renal tissue mitochondrial ATP content was assessed by the luciferin–luciferase bioluminescent assay method, as described by Hays et al. [54]. Briefly, the mitochondrial pellet samples were thawed at 10,918× *g* force for 5 min using an ultra-cooling centrifuge machine (Beckman Coulter’s ultracentrifuge, Indianapolis, IN, USA). Furthermore, 10 μL of clear supernatant was mixed with 200 μL of HEPES buffer solution. From this mixture, 200 μL of the sample was taken and mixed with 100 μL of luciferin–luciferase solution in a luminometer cuvette. The changes in bioluminescent were assessed at 562 nm by using a luminometer (Berthold Technologies GmbH and Co.KG, Bad Wildbad, Germany). The standard plot was prepared with 0, 1, 10, 100, 1000, and 10,000 nanomoles (nM) of pure ATP per mL. The value of mitochondrial ATP was expressed as nM/mg of protein.

#### 2.6.7. Estimation of Renal Mitochondrial Complex I Activity

The renal mitochondrial complex I (NADH: ubiquinone oxidoreductase) activity was assessed by using the commercial mitochondrial complex I activity colorimetric assay kit method (Abcam Sdn. Bhd., Kuala Lumpur, Malaysia). It is a useful method for the assessment of mitochondrial respiration studies in isolated mitochondria. Briefly, 2 μL of mitochondrial samples were mixed with 10 μL of complex I assay buffer. It contains 100 μL of NADH and 990 μL of complex I dye (decyl-ubiquinone) solution. The final volume of each well of microplate was made to 100 μL with complex I assay buffer. The plate was incubated at room temperature for 2 min. The conversion of decyl-ubiquinone (analog of ubiquinone; electron acceptor) to decyl-ubiquinol was achieved due to the catalytic activity of mitochondrial complex I enzyme activity. The changes in the absorbance of orange color chromogen were measured colorimetrically using a spectrophotometer (Shimadzu Corporation, Kyoto, Japan) at 600 nm wavelength. The standard plot was prepared with 0–1 mM complex I dye solution. The assay complex I activity test was performed with and without rotenone (complex I inhibitor). The mitochondrial protein concentration was measured by Lowry et al.’s method [55]. The calculation of complex I activity was performed by using the following formula:

$$Complex\;I\;Activity(\mu M/(minute/(mg\;of\;protein)=\frac{\Delta [reduced\;complex\;I\;dye\;concentration]}{\left(\Delta t\times p\right)\times D\;(mUnits/g)}$$

Here, Δ [reduced complex I dye concentration] represents the changes in reduced complex I dye concentration during the Δt period. Δt represents the changes of time, i.e., t2 − t1 in minutes. “p” indicates the mitochondrial protein concentration in μg. D represents the dilution factor. One unit of complex I activity indicates the amount of enzyme responsible for reducing the 1 μM of the dye per minute at room temperature (in pH 7.4).

### 2.7. Assessment of IS-Induced Renal Histopathological Changes

The histopathological analysis of zebrafish renal tissue was made as described by McCampbell et al. [56] with a slight modification of McKee and Wingert [57]. Briefly, in each group, two zebrafish were selected for the histopathological assessment. The zebrafish renal tissue was harvested and fixed with a neutral buffered paraformaldehyde saline (10%) solution. The renal tissue block was made with paraffin wax and embedded for cross-sectioning (5 μm thickness) under a semi-automatic cryo-microtome device. A total of five sections of each renal tissue block were analyzed. The tissue sections were analyzed and scored based on the presence of damaged proximal tubule (score 1), swollen renal glomerular tissue (score 2), vacuolar degeneration of distal tubule (score 3), and accumulation of lymphocytes and eosinophilic granule cells (score 4) as an indication of acute tubular necrosis score. The scores were converted to percentage of renal injury, i.e., 1: 0–25%; 2: 25–50%; 3: 50–75%; and 4: 75–100%. The hematoxylin–eosin staining was used for this tissue, and stained specimen slides were observed under 400× magnification. The treatment of BC-associated histopathological changes of renal tissue was compared with normal, IS, and CoQ10-treated animal tissue sections.

### 2.8. Statistical Analysis

All the results were expressed as mean ± standard deviation (SD). Data obtained from tissue biomarkers (n = 20) were statistically analyzed using one-way analysis of variance (ANOVA) followed by multiple comparison tests with Tukey’s multiple range test using Statistical Package for the Social Sciences version 25 software. A probability value of *p* < 0.05 was considered statistically significant.

## 3. Results

### 3.1. Effect of BC on IS-Induced Blood Biomarker Changes

BUN and creatinine are major hallmarks for the evaluation of renal dysfunction [58]. The exposure of IS (10 mg/L/hour/day; for 4 weeks) showed a significant (*p* < 0.05) increase in the BUN and creatinine concentrations. However, the exposure of BC (50 and 100 mg/L/hour/day; for 4 weeks) was shown to ameliorate the above IS-induced blood biomarker changes. The results were similar to the reference renoprotective agent, i.e., CoQ10 (20 mg/L/hour/day exposed for 4 weeks). CoQ10 is one of the essential components of electron carriers in the mitochondrial respiratory chain complexes I and II [59]. Furthermore, it also possesses a potential antioxidant similar to BC. We concluded that BC has a key role in the modulation of IS-induced renal injury. The changes of BC in IS-induced renal biomarkers are listed in Table 1.

### 3.2. Effect of BC in IS-Induced Renal Tissue Biomarker Changes

The lack of BCO2 activity is a marker for mitochondrial dysfunction and oxidative stress. The development of oxidative stress also indicates the reduction of SOD2 and GPX1 subsequence decrease in the GSH/GSSG ratio in mitochondria [60]. Moreover, the progress of mitochondrial inflammation, indicated by the accumulation of carbonylated proteins [61], lack of mitochondrial ATP generation, and rising complex I activity level, is an indicator of a lack of mitochondrial metabolic functions, mitochondrial energetics, and antioxidant response [62,63]. In the present study, the exposure of IS (10 mg/L/hour/day; for 4 weeks) showed a significant (*p* < 0.05) reduction of BCO2, SOD2, and GPX1 activities along with a reduction in GSH/GSSG ratio and ATP contents. It also increased the level of carbonylated protein content and complex I activity. However, the exposure of BC (50 and 100 mg/L/hour/day; for 4 weeks) was shown to ameliorate the above IS-induced renal mitochondrial biomarker changes. The results were similar to the reference renoprotective agent, i.e., CoQ10 (20 mg/L/hour/day exposed for 4 weeks). These results together suggest that BC has a key role in the prevention of IS-induced mitochondrial inflammation, mitochondrial metabolic dysfunctions, imbalance of mitochondrial energetics, and oxidative stress. The changes in BC in IS-induced renal mitochondrial biomarkers are listed in Table 2. Further, the changes of BC in IS-induced renal mitochondrial energy-dependent biomarkers are listed in Table 3.

### 3.3. Effect of BC in IS-Induced Histopathological Changes

**Figure 1:**
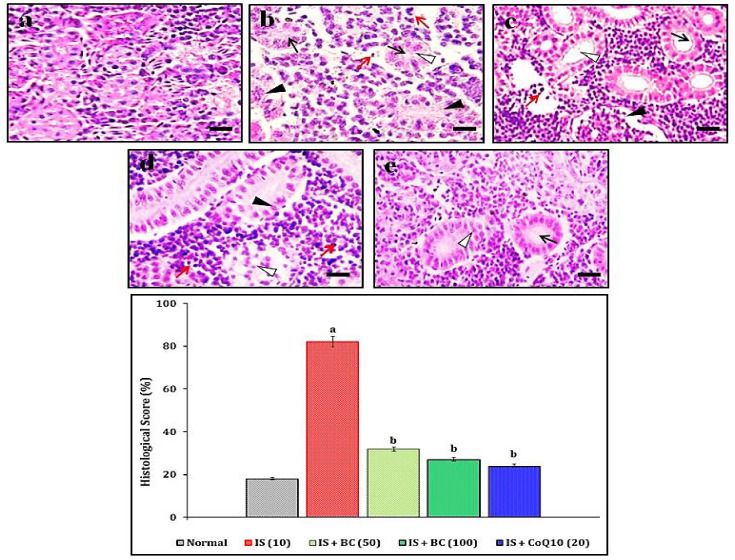
Effect of BC in IS-induced histopathological changes of renal tissue in male adult zebrafish. In each group, two zebrafish were selected for this histopathological assessment. Tissue sections were stained with Hematoxylin and eosin staining methods. (**a**–**e**) shows histological changes in renal tissue of normal control, IS (10 mg/L/hour/day; for 4 weeks), BC (50 mg/L/hour/day; for 4 weeks), BC (100 mg/L/hour/day; for 4 weeks), and CoQ10 (20 mg/L/hour/day exposed for 4 weeks) exposed groups respectively. (**a**) shows normal tissue structure. (**b**) shows the damage to the proximal tubule (black arrowhead), swollen renal glomerular tissue (white arrowhead), vacuolar degeneration of distal tubule (black arrow), and accumulation of lymphocytes and eosinophilic granule cells (red arrow). (**c**,**d**) shows mild injury in renal tissue compared to the IS-exposed group. (**e**) shows the potential renoprotective actions as similar to normal tissue. The semi-quantitative score of histopathological changes was also evidenced by attenuation of BC and CoQ10 actions against the IS exposure. Symbol in the bar-graph of histological score expressed ^a^ *p* < 0.05 vs. normal group. ^b^ *p* < 0.05 vs. IS control group. Microscopic examinations were performed under a 400× magnification, scale bar 35 µm.

The histological changes in zebrafish renal tissue in normal control animals show no changes in the proximal tubule, renal glomerular tissue, distal tubule, lymphocytes, and eosinophilic granule cells structure, whereas the IS (10 mg/L/hour/day; for 4 weeks) were shown to have potential changes in renal tissue, i.e., damage of proximal tubule, swelling of the renal glomerulus, vacuolar degeneration of the distal tubule, and accumulation of lymphocytes and eosinophilic granule cells. The histopathological score was also increased significantly in the IS-exposed group. However, the exposure of BC (50 and 100 mg/L/hour/day; for 4 weeks) showed the potential amelioration of histopathological changes in renal tissue in adult zebrafish with a reduction of the histopathological score. The results were similar to the reference renoprotective agent, i.e., CoQ10 (20 mg/L/hour/day exposed for 4 weeks). We concluded that BC possesses preventive action against IS-induced mitochondrial inflammation-associated histopathological changes. The changes were observed under 400× magnifications (scale bar: 35 µm). The effects of BC in IS-induced histopathological changes are depicted in Figure 1.

## 4. Discussion

The present study results revealed that the exposure of IS (10 mg/L/hour/day for 4 weeks) showed a significant (*p* < 0.05) increase in BUN and creatinine concentrations; a reduction of BCO2, SOD2, and GPX1 activities; along with a reduction in GSH/GSSH ratio and ATP contents. Further, IS induces the accumulation of carbonylated proteins and complex I activity levels in isolated mitochondria. Furthermore, IS exposure is also shown to significant histopathological changes in renal tissue. Moreover, the 4-week exposure to BC (50 and 100 mg/L/hour/day) and CoQ10 (20 mg/L/hour/day) ameliorated the above IS-induced renal tissue biomarkers and histopathological changes.

Mitochondrial BCO2 activity is essential for the conversion of natural carotenoids by different carotenoid oxygenase enzymes, i.e., β-carotene oxygenase-1 (BCO1; located in the cytoplasm) and BCO2 (located in the inner mitochondrial membrane) [64]. BCO1 and BCO2 contribute to the metabolic conversion of BC, whereas BCO2 contributes to BC catabolism in the excessive doses of BC presence [65,66]. BCO2 is also reported to produce protective action against carotenoid-induced mitochondrial inflammation [67]. The *BCO2* gene is tightly associated with the accumulation of carotenoids like BC [68]. The combined action of BCO2 and BC is expected to produce better therapeutic actions [69,70,71]. BCO2 possesses renoprotective action via the cell-specific expression of the β-carotene 9′, 10′-monooxygenase enzyme in human tissue [72]. Similar results were observed in the present study, in which BC produced renoprotective action via the activation of the BCO2 enzyme and related protein expression.

There are three isoforms of SODs that are widely distributed in cellular systems. The isoform of SOD2 is specifically located in the inner mitochondrial matrix [73]. All three isoforms contribute to the regulation of mitochondrial membrane potential and mitochondrial electron transport chain functions [74]. Moreover, the antioxidative defense enzymes, i.e., SOD2 and GPX1, contribute to the progression of renal dysfunction [75]. Experimentally, the lack of GPX1 and SOD2 genes affects the tissue mitochondria, leading to oxidative stress in mice [76]. Overall, SOD2 converts oxygen radicals into hydrogen peroxide, causing mitochondrial dysfunctions [77]. The antioxidative enzymes, i.e., superoxide dismutase (SOD2) and glutathione peroxidase (GPX1), produce the first line of defense actions against free radicals and pathogens [78]. In pathological conditions, there is evidence to reduce the SOD2 and GPX1 enzymes. The association of GPX1 and SOD2 contributes to ochratoxin (OTA)-induced nephrotoxicity and Balkan endemic nephropathy [79]. Recent literature reports have also evidenced that SOD2 and GPX1 activity levels are reduced in organophosphorus pesticides, i.e., chlorpyrifos-associated organ toxicity [80]. Moreover, uremic toxin, i.e., IS, is known to reduce the SOD2 and GPX1 activities during early vascular aging and the calcification process [81]. Similar results are observed in IS-induced renal dysfunction of male adult zebrafish. Further, BC prevents mitochondrial inflammations via the activation of GPX1 and SOD2 enzyme activities [82]. Our present data also evidenced that BC prevents IS-induced mitochondrial inflammation by the accumulation of carbonylated proteins.

Mitochondria are the powerhouse of every cell and generate ATP energy [83]. The production of mitochondrial ATP is mainly due to the modification of sulfhydryl redox proteins with the influence of nicotinamide adenine dinucleotide and flavin adenine dinucleotide generation and free electron flow of the electron transport chain process [84]. Mitochondrial ATP generation is mainly through mitochondrial complex I to IV enzyme functions. The mitochondrial complex I functions is responsible for the oxidative phosphorylation-mediated ATP production and maintenance of a balanced mitochondrial respiratory chain [85]. In this condition, the mitochondrial Krebs cycle is involved in the oxidization of NADH for electron transfer for the reduction of ubiquinone to ubiquinol [86]. The alteration of the mitochondrial bioenergetics process is widely involved in the progression of CKD via oxidative stress and mitochondrial dysfunction [87]. The reduced form of glutathione (GSH) is an endogenous antioxidant substance [88]. In pathological conditions, GSH was oxidized and generated the GSSH form of glutathione, leading to a reduction in the GSH/GSSH ratio due to the depletion of GSH and subsequent accumulation of free radicals. It widely indicates mitochondrial oxidative stress and lack of mitochondrial functions. In zebrafish, the reduction of GSH/GSSG was observed in bacterial lipopolysaccharide-induced mitochondrial dysfunctions [89,90].

The mitochondrial GSH plays a key role in supporting the efficient functions of mitochondria [91]. BC administration is known to reduce oxidative stress via the enhancement of mitochondrial GSH and ATP levels, leading to improved mitochondrial respiration [92,93]. BC also shows potential preventive action against ischemia/reperfusion-induced renal injury [39]. BC is metabolically converted by mitochondrial BCO2 action, which leads to a lack of quantity of BCO2 for constitutive mitochondrial functions and develops oxidative stress [38]. In contrast, carotenoids also possess enzymatic and non-enzymatic cellular responses against oxidative stress [94]. This could be the activation of mitochondrial GPX1 and SOD2 enzyme activities and the elimination of reactive oxygen species [95]. Low-molecular-weight antioxidants like GSH, vitamin E, and ubiquinone are known to improve mitochondrial functions in various disorders [84]. Antioxidants like BC are known to enhance the intracellular glutathione level [96]. This could be a major factor in the amelioration of BC in IS-induced mitochondrial dysfunctions. Similarly, CoQ10 is also known to boost mitochondrial GSH contents due to its key functions in the mitochondrial respiratory chain [97]. Experimentally, CoQ10 alleviates tacrolimus-induced renal mitochondrial dysfunction [98]. The present results also revealed that BC and CoQ10 possess multiple roles in the prevention of IS-induced renal dysfunction in zebrafish.

Crucially, the above literature report and present study results revealed that the exposure to IS attenuates renal dysfunctions, as proven in the histopathological observation. BC has greater scavenging efficacy of reactive oxygen species (singlet oxygen). Experimentally, BC is evidenced to protect the renal tissue via enhancement of GSH, SOD, and CAT levels in renal tissue [99]. Moreover, the administration of BC attenuates the renal carbonylated protein-associated tissue damage [100]. BC and other antioxidants are known to regulate the mitochondrial functions in chronic kidney disorder conditions [101]. Similar results were also observed in the present study. This is mainly due to the BC and BCO2-associated regulation of mitochondrial SOD2, GPX1, GSH, and ATP content levels in renal tissue. Based on this research outcome, BC can be used for renal disorders, especially in CKD patients. However, the limitation of this study was carried out in lower vertebrate models. It can be tested in higher vertebrate models before entering into phase-1 clinical trials. Therefore, more extensive studies are required to explore the complete therapeutic potential of BC action in chronic renal disorder conditions. Our research team has taken a key interest in exploring these actions of BC in the management of renal disorder in higher vertebrate animal models, and this investigation is in progress.

## 5. Conclusions

**Figure 2:**
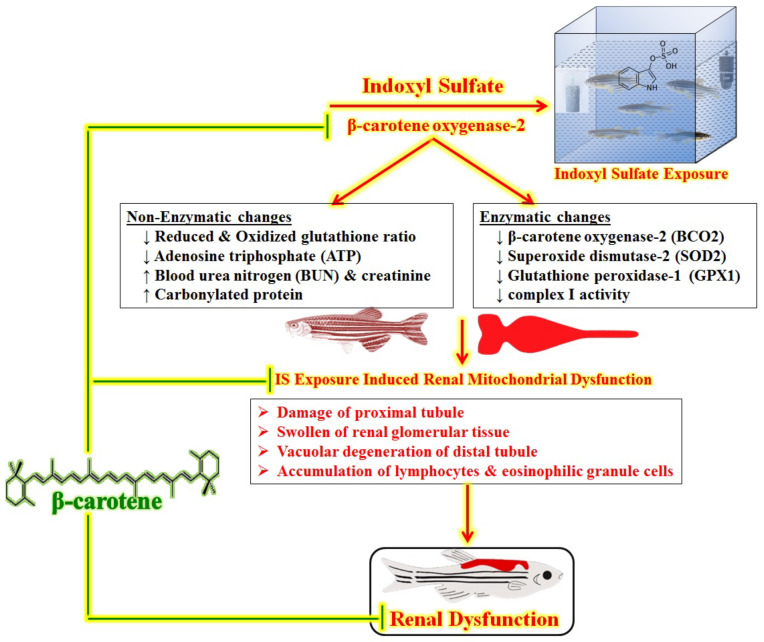
Summary of BC actions on IS-associated mitochondrial-dependent renal dysfunction. The exposure to IS caused potential renal damage via elevation of BUN, creatinine, and carbonylated protein concentration and reduction of BCO2, SOD2, GPX1, and complex I activities, which led to a decrease in the GSH/GSSG ratio and ATP levels. BC attenuates the IS-induced pathological changes in renal disorder. It indicates that BC has preventive action against renal injury via inhibition of BCO2-associated mitochondrial inflammation and dysfunction.

We conclude that exposure to BC prevents IS-induced renal dysfunction in adult male zebrafish. BC improves the IS-induced mitochondrial inflammation and dysfunctions via the enhancement of BCO2, SOD2, GPX1, and complex I activity levels and the GSH/GSSH ratio, and reduces the carbonylated proteins GSH/GSSH ratio and ATP contents in renal tissue. The deficiency of BCO2 is one of the major contributors to the progression of oxidative stress, mitochondrial dysfunction, and renal tissue damage. Hence, we concluded that the natural carotenoid, i.e., BC can be used for the management of uremic toxin (IS)-induced renal dysfunctions due to its multi-targeted actions, including BCO2 activation. The summary of BC actions on IS-associated mitochondrial-dependent renal dysfunction has been illustrated in Figure 2.

## Figures and Tables

**Table 1:** Effect of BC in IS-induced blood biomarkers changes.

Groups	BUN (mM/L)	Creatinine (µM/L)
Normal	6.24 ± 0.4	20.19 ± 0.7
IS (10)	12.83 ± 0.9 ^a^	39.72 ± 0.8 ^a^
IS + BC (50)	8.17 ± 0.8 ^b^	28.81 ± 0.5 ^b^
IS + BC (100)	7.36 ± 0.6 ^b^	25.71 ± 1.1 ^b^
IS + CoQ10 (20)	6.94 ± 0.3 ^b^	21.26 ± 0.9 ^b^

Data were expressed as mean ± SD, n = 20 zebrafish per group. Digits in parenthesis indicate the dose mg/L/hour/day for 4 weeks of exposure. ^a^ *p* < 0.05 vs. normal group. ^b^ *p* < 0.05 vs. IS control group. Abbreviation: BC, beta-carotene; BUN, blood urea nitrogen; CoQ10, coenzyme Q10; and IS, indoxyl sulfate.

**Table 2:** Effect of BC in IS-induced renal biomarkers changes.

Groups	BCO2 (nM/mg of Protein)	SOD2 (ng/mg of Protein)	GPX1 (U/mg of Protein)	GSH/GSSG Ratio	Carbonylated Proteins (nM/mg of Protein)
Normal	1.98 ± 0.2	49.29 ± 2.1	3.61 ± 0.8	8.23 ± 0.6	1.91 ± 0.9
IS (10)	0.13 ± 0.4 ^a^	13.18 ± 1.5 ^a^	1.02 ± 1.2 ^a^	1.79 ± 1.2 ^a^	10.54 ± 0.8 ^a^
IS + BC (50)	1.06 ± 0.3 ^b^	31.47 ± 1.9 ^b^	2.71 ± 0.7 ^b^	4.01 ± 0.8 ^b^	4.59 ± 0.7 ^b^
IS + BC (100)	1.37 ± 0.5 ^b^	37.82 ± 1.1 ^b^	3.14 ± 0.9 ^b^	3.27 ± 0.6 ^b^	3.87 ± 0.4 ^b^
IS + CoQ10 (20)	1.63 ± 0.6 ^b^	45.39 ± 1.3 ^b^	3.45 ± 0.6 ^b^	2.36 ± 0.9 ^b^	2.61 ± 0.3 ^b^

Data were expressed as mean ± SD, n = 20 zebrafish per group. Digits in parenthesis indicate the dose mg/L/hour/day for 4 weeks of exposure. ^a^ *p* < 0.05 vs. normal group. ^b^ *p* < 0.05 vs. IS control group. Abbreviation: BC, beta-carotene; BCO2, β-carotene oxygenase-2; CoQ10, coenzyme Q10; GPX1, glutathione peroxidase-1; GSH/GSSG, reduced and oxidized glutathione; IS, indoxyl sulfate; and SOD2, superoxide dismutase-2.

**Table 3:** Effect of BC in IS-induced renal mitochondrial energy-dependent biomarkers changes.

Groups	ATP (nM/mg of Protein)	Complex I Activity (µM/Minute/mg of Protein)
Normal	5.73 ± 0.6	47.28 ± 1.2
IS (10)	1.26 ± 0.3 ^a^	12.04 ± 1.4 ^a^
IS + BC (50)	3.47 ± 0.8 ^b^	36.56 ± 0.9 ^b^
IS + BC (100)	4.62 ± 0.5 ^b^	40.28 ± 1.5 ^b^
IS + CoQ10 (20)	5.41 ± 0.7 ^b^	43.91 ± 1.3 ^b^

Data were expressed as mean ± SD, n = 20 zebrafish per group. Digits in parenthesis indicate the dose mg/L/hour/day for 4 weeks of exposure. ^a^ *p* < 0.05 vs. normal group. ^b^ *p* < 0.05 vs. IS control group. Abbreviation: ATP, adenosine triphosphate; and Complex I Activity, mitochondrial NADH: ubiquinone oxidoreductase activity.

## Data Availability

Data are contained within the article.
